# What factors determine brand communication? A hybrid brand communication model from utilitarian and hedonic perspectives

**DOI:** 10.3389/fpsyg.2022.958863

**Published:** 2023-01-18

**Authors:** Lingzhi Brian Fang, Mingzhen Liu, Liu Tang

**Affiliations:** ^1^School of Journalism, Fudan University, Shanghai, China; ^2^School of Management, Fudan University, Shanghai, China

**Keywords:** hybrid brand communication mechanism, customer engagement, customer psychological contract, utilitarian and hedonic functions in brand communication, information function

## Abstract

**Introduction:**

With the advancement of new media, brand communication has been taken into consideration by lots of firms. Apparently, customer affection plays a significant role in brand communications, though few studies have determined how the twofold of information function works in this communication mechanism. Based on this research gap and practical background, this paper proposes a hybrid model of communication comprising the utilitarian and hedonic aspects.

**Methods:**

For this study, 575 questionnaires were collected, followed by the structural equation modeling of the derived data to test the research model.

**Results:**

The results of statistical analysis show that the brand communication can be improved in terms of both utilitarian and hedonic aspects. Moreover, psychological contract and customer engagement play a chain mediation role in this mechanism.

**Discussion:**

These findings contribute to the research of brand communication mechanism in digital era. Likewise, the findings offers several practical implications to the brand management.

## 1. Introduction

Brand communication in the digital era has been reshaped by new media platforms (Voorveld, 2019), which changed the idea of how brands communicate on social media. Based on prior studies on brand communication, it has been reached into the consensus that the brand function determines the spreading and communication effect of a brand (Lynch and de Chernatony, 2004; Steinmann et al., 2015). Thus, firms are always focusing on the effects exerted by the utilitarian function brought by a brand.

There are not only utilitarian functions provided by brands. Current realities indicate that psychological aspects of customers have begun to lie at the center of studies on customer behavior. However, to date, many firms still insist that brands can only deliver utilitarian functions to customers, thus, ignoring customers' spiritual aspect, which has been criticized by academics (Lynch and de Chernatony, 2004; Ham et al., 2019). Thus, consistent with the relational marketing era coming, it is of great necessity that the hedonic function, that is, spiritual aspects of brands, should be taken into consideration instead of the utilitarian function of brands (Choi and Choi, 2014). Such revealing on brand communication demonstrates that brand communication in this modern era is a hybrid mixture of utilitarian and hedonic functions (Underwood and Klein, 2002; Wijaya, 2013; Uzunoglu and Kip, 2014).

Moreover, the arousal of customer relationship management requires scholars to focus on the customer's psychological aspect in brand communication (Sullivan et al., 2020). Unfortunately, few researchers have investigated how utilitarian and hedonic functions simultaneously shape brand communication. There are existed few studies detecting this psychological role in the process of brand communication. In addition, prior studies have shown the roles of utilitarian or hedonic aspects in brand communication desperately, whereas few studies have integrated these two aspects into one consideration (Simon, 2017; Ham et al., 2019). This gives this study the main research question: how can we process a satisfying brand communication from the utilitarian and hedonic perspectives with linkage to the customer relationship?

Based on mentioned research gap and the practical background, this study poses a hybrid model of brand communication from the perspectives of a two-fold information function. Specifically, this hybrid model is focused on the utilitarian and hedonic functions of information. Relying on customer relationships, the customer psychological contract and customer engagement are proposed for the mediators. This study shows that brand communication can be strengthened both from utilitarian and hedonic perspectives. According to the results, the psychological aspects, that is, the hedonic function, take precedence over the utilitarian function in this communication mechanism. The results contribute to the studies on customer psychology and behavior.

This study is organized as follows. In the first part, the related literature is reviewed in order to introduce the theoretical background and then develop the research model. Hypotheses are also posed in this part. The second part mainly emphasizes the method. In this study, a survey was used to collect the data for testing the model. Then, structural equation modeling (SEM) was applied to evaluate the relationship among all the variables. At the end of this study, based on the results of data processing, we further discussed the results and proposed several theoretical contributions and practical implications. More specifically, the proposition on the hybrid model of brand communication contributes to the research of brand communication, especially if it is from a novel perspective of information functions, that is, the utilitarian and hedonic functions. Furthermore, it offers several practical implications for making branding strategies in the post-truth era.

## 2. Theoretical background and research model

### 2.1. Literature review

#### 2.1.1. Brand communication: A perspective of information function

The rise of research on brand communication flourished because of the popularity of social media and first began in a top marketing research journal in 2008 (Voorveld, 2019). This fact suggests that brand communication is linked closely to the development of media. Based on this, as for the definition of brand communication, prior studies demonstrated that brand communication is any piece of brand-related communication “distributed *via* social media” (Yang and Battocchio, 2020; Arya et al., 2022). Thus, this definition can lead to an observation that brand communication is a reflection of the relationship between internet users and the brand. Furthermore, it can be conducted that brand communication is related to the customer–brand relationship (Sharma and Varki, 2018; Dewnarain et al., 2019; Youn and Jin, 2021).

Many scholars have brought up a large number of research regarding brand communication from diversified perspectives. According to Bergkvist et al. (2012), brand communication effect has been influenced by the headlines of an ad. The result of this research showed that a complete headline is beneficial for brand communication. Simultaneously, communication style may have an impact on brand communication. According to Steinmann et al. (2015), communication style influences the attitude and recommendation of the brand. Simon (2017) has found that communal media gratification contributed more to brand gratification. This finding was significant for brand communication from the perspective of media. As a result, prior studies have revealed the fact that brand communication is a complex concept linked with information and media; however, few studies take the viewpoint of information function into the research of brand communication.

Obviously, it is of significant necessity to identify the information foundation on brand communication, due to brand communication being an information process (Voorveld, 2019; Yang and Battocchio, 2020). Thus, several studies regarding the functions of information were reviewed as follows. First, the information function is known for the utilitarian and hedonic perspectives. As for utilitarian function, prior research has focused on information usefulness as a utilitarian feature of information. According to Luo et al. (2018), information usefulness is regarded as an extension that has an impact on the intention and behavior of individuals. Thus, various studies show that information usefulness is the information that is perceived as valuable by readers. This finding depicts the utilitarian aspect of information (Saeed and Abdinnour-Helm, 2008; Gottlieb, 2012; Ham et al., 2019). Moreover, as different forms of information presentation may activate the psychological aspect of the customer itself, information also provides a hedonic function. There are several examples serving as evidence that image may be one of the most important formations of information to trigger the sentiment or enjoyment of customers. According to its definition, an image can provide a sensory experience for customers (O'Shaughnessy and O'Shaughnessy, 2002; Wijaya, 2013; A-Qader et al., 2016; Japutra et al., 2021). This may lead the customer to behave more psychologically, and as such, there is a multitude of reviews with positive sentiments on Instagram (Yu and Egger, 2021; Rejeb et al., 2022). This fact shows that perceived image can trigger the psychological aspect of customers, which reflects the hedonic function in brand communication.

Overall, the information feature of brand communication reveals the necessity to discuss brand communication from the perspective of information function. However, few studies identify this process from such perspectives. Consistent with studies into information function from utilitarian and hedonic perspectives, this theoretical viewpoint offers a research gap for us to fill up on brand communication.

#### 2.1.2. Building the brand communication mechanism from utilitarian and hedonic perspectives: The role of customer relationship

Although two aspects of information function have been revealed, in order to detect a more explicit mechanism of communication on how information function works, we have focused on customer relationships.

According to prior related studies, customer psychological contract and customer engagement have lied at the center of the research into customer–brand relationships (Gillani et al., 2021; Lin et al., 2021; Asante et al., 2022; Sim et al., 2022). When it comes to customer psychological contract, existing research has proposed that the psychological contract was first proposed in the studies of organizational behavior and then defined in a relatively straightforward manner, stressing individual and organizational perceptions of obligations while also highlighting the stability of their connection (Robinson et al., 1994; Coyle-Shapiro et al., 2019). Above all, a psychological contract is the manifestation of a spiritual contract. Moreover, the psychological contract has been extended to a variety of contexts in recent studies and is no longer limited to organizational settings. Customer psychological contract is the extension of the psychological contract from the organizational scenario to the marketing scenario, which specifically studies the relationship between the consumer and the enterprise (Bi, 2019; Tomprou and Lee, 2022). Previous studies have shown that transactional and relational psychological contracts are two types of psychological contracts (Coyle-Shapiro et al., 2019). In summary, the customer psychological contract symbolizes a process of deepening the relationship between subjects (e.g., individuals) and objects (e.g., the media). The two categories of psychological contract reveal that this concept typically describes the relationship from both utilitarian and hedonic perspectives (Ozturk et al., 2016).

In addition, with the transformation of media technology, we are entering the “engagement” era (Ferrer-Conill et al., 2021). Consistent with the acceleration of customer participation evolving into customer involvement, numerous studies have still classified these relationships as having no profound bonds (Myrick and Erlichman, 2020; Yamamoto et al., 2020; Barari et al., 2021).

Thus, the proposition of customer engagement found by researchers aimed to identify this profound connection. This novel relationship has resulted in customer engagement both in customer psychology and behavior. Thus, customer engagement is a typical manifestation of a deeper customer relationship (Brodie et al., 2019). Undoubtedly, the permeation of customer engagement has a typical impact on all customers (Gummerus et al., 2012; Meire et al., 2019). Kilger and Romer (2007) confirmed that customer engagement impacts consumer purchase intention. Lin et al. (2021) stated that the popularity of live broadcasting is directly related to customer engagement. Although prior studies have begun to explore the impact of customer engagement (Pentina et al., 2018), few have explored the role and impact of this novel relational formation in brand communication.

Building upon the new media has brought an evolved relationship between customers and media; brand communication has thus been shifted and influenced by this relationship transition (Youn and Jin, 2021). In addition to information function studies, this relational transition demonstrates that brand communication in the digital age may be shifted by psychological and behavioral aspects of customers (Park and Ha, 2016; Steinhoff et al., 2019). This analysis has offered a research gap for this study; therefore, we are aiming to figure out the effect of customer relationship exerting on brand communication.

### 2.2. Hypotheses deduction

According to the literature on psychological contracts mentioned before, there are two ways to construct a customer psychological contract from the perspectives of transaction and relation, which reflects the two-fold of information function (Ham et al., 2019; Yuan et al., 2022). First and foremost, from the utilitarian perspective, customers always seek out the value of information. From this standpoint, information value provided by the brand can facilitate customers to make useful decisions. Thus, customers could have “perceived quality” through the information value. This may lead to the construction of a psychological contract from a transactional aspect (Luo et al., 2018; Zhao et al., 2020). In contrast, there is also a relational and psychological way for customers to build up the customer psychological contract. In terms of customers, brand image can offer them a more hedonic and sentimental experience (A-Qader et al., 2016; Park and Kim, 2022). As a result, a brand image can have more influence on the customers' psychological aspect from the hedonic perspective. Overall, the compared hypotheses shall be posed and identified. Therefore, Hypothesis 1 and its sub-hypotheses can be proposed, as follows, in accordance with the preceding analysis.

H1. Different forms of information function lead to the construction of the customer psychological contract in both utilitarian and hedonic ways.
H1a. The perceived quality has more impact on the construction of a transactional psychological contract rather than a relational psychological contract *via* the utilitarian aspect.
H1b. The brand image has more impact on the construction of a relational psychological contract rather than a transactional psychological contract *via* the hedonic aspect.

In this study, however, considering that the customer psychological contract can be constructed by information from both utilitarian and hedonic perspectives, it can be deduced that the customer psychological contract can facilitate the customer relationship to march into the status of engagement (Kumar et al., 2019). According to current research, the psychological contract can be categorized into two aspects; one is behavioral, and the other is psychological (Gillani et al., 2021; Asante et al., 2022). When it comes to customer engagement, two constructive ways are to be recognized. Based on the two aspects of information function, the behavioral aspect of the psychological contract represents the utilitarian aspect. Similarly, the psychological aspect of the psychological contract represents the hedonic aspect. That is, the transactional psychological contract can be linked to the utilitarian aspect, and the relational psychological contract to the hedonic aspect (Tomprou and Lee, 2022; Yuan et al., 2022). Specifically, from the utilitarian perspective, the transactional psychological contract has played a critical role. Similarly, the relational psychological contract is necessary for customer engagement from a hedonic perspective. Once customer engagement has been activated, brand communication will be improved. Consequently, Hypothesis 2, with its sub-hypotheses, and Hypothesis 3 were developed here as follows.

H2. The customer psychological contract facilitates the achievement of customer engagement.
H2a. The transactional psychological contract facilitates the achievement of customer engagement *via* the utilitarian aspect.
H2b. The relational psychological contract facilitates the achievement of customer engagement *via the* hedonic aspect.
H3. Customer engagement improves brand communication.

In order to build the whole hybrid brand communication model, all the factors should be taken into consideration. To begin with, as for the utilitarian aspect, it can be concluded that perceived quality could benefit the construction of the transactional psychological contract. Furthermore, the transactional psychological contract facilitates the establishment of customer relationship and then lead to achieving customer engagement, ultimately improving brand communication *via* the utilitarian aspect. In addition, from the hedonic perspective, the brand image assists the construction of the relational psychological contract, thus resulting in customer engagement. Therefore, brand communication can be improved by this impact mechanism in a hedonic way.

In summary, this two-fold aspect of customer psychological contract, as well as customer engagement, offers a chain mediation to improve brand communication from both utilitarian and hedonic perspectives. Based on this, this study presents Hypothesis 4 and its sub-hypotheses, listed later.

H4. The psychological contract and customer engagement act as chain mediators in the mechanism of improving brand communication from both utilitarian and hedonic perspectives.
H4a. The transactional psychological contract and customer engagement play a chain mediating role in the mechanism of improving brand communication *via* the utilitarian aspect.
H4b. The relational psychological contract and customer engagement play a chain mediating role in the mechanism of improving brand communication *via the* hedonic aspect.

### 2.3. Research model

**Figure 1:**
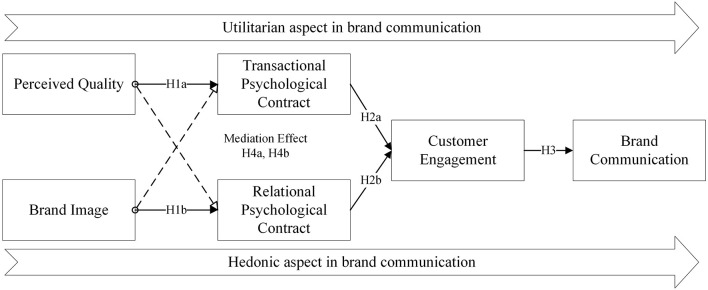
Research model of hybrid communication in utilitarian and hedonic aspects.

Consistent with the literature review mentioned before, it is of significance to figure out the effects of information function exerted in brand communication. The two-fold of information function has a different role in brand communication. Based on this, Figure 1 shows the research model developed in this study.

Specifically, to begin with, perceived quality facilitates the construction of the transactional psychological contract and leads to customer engagement, which improves brand communication from the utilitarian perspective. Moreover, the perceived image assists in building up the relational psychological contract hedonically. Then, brand communication will be improved as a result of customer engagement being achieved from the hedonic perspective. As for mediators, transactional psychological contract and customer engagement play a chain mediating role in this mechanism from the utilitarian perspective. Similarly, relational psychological contract and customer engagement act as hedonic chain mediators.

## 3. Method

This study has mainly relied on a survey method. All participants were required to finish the questionnaire for inclusion in the study. Therefore, we distributed our questionnaires through social media platforms, in which a third-party survey corporation was entrusted for distribution.

At the beginning of the questionnaire, all participants were requested to fill in a brand they were familiar with. The remaining questions were required to be finished in accordance with the brand they filled in.

Specifically, this questionnaire was designed in four parts. In the first part, after filling in the brand, there are questions about perceived quality and brand image. The second part is items of customer psychological contracts and customer engagement. The third part is for testing brand communication. The last part of the questionnaire was designed to collect the demographic data of participants as control variables. All parts of the questionnaire adopted a 5-point Likert scale for responses (from “1 = strongly disagree” to “5 = strongly agree”).

### 3.1. Survey procedure

Due to the whole survey procedures were taken place in China from January 2021 to June 2021, it is necessary for our researchers to take the difference in the research context into consideration. In order to ensure the reliability and validity of the results of this study, it was decided that all the questionnaires be translated into Chinese and that local versions of the instruments be adopted as much as possible. This study tries to ensure that the instruments adopted by this research closely follow those already published in high-level domestic and international academic journals.

To ensure the suitability of the instrument, this study first introduced a pre-test for all questionnaires. Approximately 10 individuals were requested to finish and examine the instruments. After this small-scale testing, we revised the instrument based on the result of the data pre-test.

After that, a large-scale survey was conducted to verify the model. At the beginning of the instrument, participants were required to fill in a brand with which they were always communicating. Then, they were asked to finish the survey based on what they had filled in. The instrument was distributed by ourselves and the third-party survey corporation as well. Overall, a total of 575 valid questionnaires were collected in this study after setting up reverse question items, establishing question items in the same direction, and removing questions in which the answer time was too short (*t* < 180 s) and those with a series of identical answers. These 575 questionnaires include 325 from the “snowball” collection and 250 from the third-party survey corporation. The psychometric properties of the sample are displayed in Table 1.

**Table 1:** Psychometric properties of the survey.

**Category**	**Characteristics**	***N***	**%**
Gender	Male	261	45.4
	Female	314	54.6
Age	Under 18	1	0.2
	18–25	103	17.9
	26–30	139	24.2
	31–40	185	32.2
	41–50	114	19.8
	51–60	29	5.0
	Above 60	4	0.7
Education	High school or lower	28	6.8
	Junior college	99	17.0
	Bachelor's degree	435	52.5
	Master's degree or higher	53	33.7
Occupation	Government agency or public institution	110	19.1
	Enterprise employee	302	52.5
	Individual industrial and commercial households	31	5.4
	Students	77	13.4
	Other	55	9.6
Salary (monthly)	Under 1,000 CNY	30	5.2
	1,000 CNY−3,000 CNY	62	10.8
	3,000 CNY−5,000 CNY	106	18.4
	5,000 CNY−8,000 CNY	159	27.7
	Above 8,000 CNY	218	37.9

*N* = 575; this study will assign a value to the control variable, which has listed later. Gender: 1 = male; 0 = female. Age: 1 = under 18; 2 = 18~25; 3 = 26~30; 4 = 31~40; 5 = 41~50; 6 = 51~60; 7 = above 60. Education: 1 = high school or lower; 2 = junior college; 3 = bachelor's degree; 4 = master's degree or higher. Occupation: 1 = government agency or public institution; 2 = enterprise employee; 3 = individual industrial and commercial households; 4 = students; 5 = other. Salary (monthly): 1 = under 1,000 CNY; 2 = 1,000 CNY−3,000 CNY; 3 = 3,000 CNY−5,000 CNY; 4 = 5,000 CNY−8,000 CNY; 5 = above 8,000 CNY.

In the large-scale survey, 54.6% of the survey participants were female participants (*N*_*female*_ = 314, *M*_*sex*_ = 0.454, *SD*_*sex*_ = 0.208), which is slightly more than for male participants. It also can be observed that people aged 31–40 accounted for 32.2% of the total sample that paid more attention to this research (*M*_*age*_ = 3.715, *SD*_*age*_ = 0.0486). At the same time, the majority of these participants, 52.5% of the whole sample, have a bachelor's degree (*M*_*edu*_ = 2.93, *SD*_*edu*_ = 0.0343). People from companies accounted for 52.5% of the entire sample (*M*_*job*_ = 2.417, *SD*_*job*_ = 0.506). Finally, persons with a salary exceeding 8,000 CNY per month made up 37.9% of the whole sample (*M*_*salary*_ = 3.823, *SD*_*salary*_ = 0.05).

### 3.2. Measurement of variables

As for the measurement of variables, this research first adopted suitable items to test all six variables. Furthermore, in order to identify whether these items are fit for this research context, confirmatory factor analysis (CFA) and reliability analysis were employed for each measurement of these variables. Table 2 shows the reliability and convergent validity results of all the above-mentioned variables in the research model.

**Table 2:** Reliability and convergent validity result for measurements of variables.

**Validity and reliability index**		**Perceived quality**	**Brand image**	**Transactional psychological contract**	**Relational psychological contract**	**Customer engagement**	**Brand communication**
CFA index	CMIN/DF	–	3.33	1.962	2.91	3.7	–
	RMR		0.014	0.016	0.011	0.029	
	TLI		0.965	0.988	0.995	0.944	
	GFI		0.985	0.993	0.973	0.958	
	CFI		0.981	0.994	0.991	0.96	
	RMSEA		0.064	0.041	0.058	0.069	
Cronbach's α		0.749	0.818	0.81	0.71	0.858	0.676
Number of items		2	6	5	4	10	2

*N* = 575; CMIN/DF represents $\frac{\chi^{2}}{df}$; RMR, the root-mean-square residual; TLI, the Tucker–Lewis index; GFI, goodness-of-fit index; CFI, the comparative fit index; RMSEA, root-mean-square error of approximation.

First, we attempted to find suitable items for measuring perceived quality and brand image. For measuring perceived quality, this study refers to the items of perceived quality provided by Yoo and Donthu (2001) and Snoj et al. (2004). Since this variable was measured by only two items, we just assessed its reliability and validity based on Cronbach's α, which was 0.749. In terms of brand image, there were six items that refer to measure this variable, according to Keller (1993) and Yoo and Donthu (2001). We then utilized CFA and reliability test to identify whether this measurement is fit for this research. The whole results show that this variable is suitable for this study.

Moreover, in terms of the variable of the psychological contract, we have referred to the research regarding employee psychological contract (Coyle-Shapiro et al., 2019) and customer psychological contract (Kingshott and Pecotich, 2007). After the interview of 30 people and grounding theory, five items for the transactional psychological contract and four items for the relational psychological contract have been generated. After CFA, the results of these two concepts show it has a great degree of validity and reliability. As for customer engagement, the measurements of this concept referred to some studies regarding social media engagement (Brown et al., 2022) and customer engagement (Brodie et al., 2013; Kumar et al., 2019). The pre-test results for testing customer engagement show that the measurement of this variable possesses a great degree of validity and reliability for this study.

Finally, according to some studies on communication and brand communication (Voorveld, 2019; Harrison and Windeler, 2020), the measurement of brand communication was considered based on two items from the perspective of personal influence. The brand communication passed the validity and reliability test based on its Cronbach's α of 0.676.

### 3.3. Data pre-test

#### 3.3.1. Data quality

Since questionnaires were gathered in a variety of ways, an independent-sample *t*-test was conducted on the data from two distinct sources to determine whether the questionnaire collection method influenced the analytical results. The results of the independent-sample *t*-test are presented in Table 3. The test reveals that the alternative ways of questionnaire collection had no impact on the study results. In order to avoid common method biases (CMBs) in this survey, Harman's single factor test was used to test sample data in the statistical control, effectively also avoiding common approach deviations in procedural control. It can be observed from the analytical results that the CMB of this survey is within an acceptable range (*Var%*_*component* 1_ = 30.012, *Cultimative%*_*component* 1_ = 30.012).

**Table 3:** Independent-sample *t*-test for variation in the methods for instruments.

**Constructs**		**Levene's test for equality of variances**		***T*** **-test for equality of means**			
		***F***	**Sig**.	***T***	**Sig. (two-tailed)**	**Mean difference**	**SD**
Customer engagement	Equal variances assumed	0.035	0.852	0.141	0.89	0.01	0.04
	Equal variances not assumed	–		0.14	0.89	0.01	0.04
Transactional psychological contract	Equal variances assumed	0.006	0.939	−1.01	0.31	−0.06	0.06
	Equal variances not assumed	–		−1.011	0.31	−0.06	0.06
Relational psychological contract	Equal variances assumed	0.421	0.517	0.966	0.33	0.04	0.04
	Equal variances not assumed	–		0.964	0.34	0.04	0.04
Brand image	Equal variances assumed	0.302	0.583	0.953	0.34	0.04	0.04
	Equal variances not assumed	–		0.941	0.35	0.04	0.05
Perceived quality	Equal variances assumed	0.95	0.33	1.035	0.30	0.05	0.05
	Equal variances not assumed	–		1.039	0.30	0.05	0.05
Brand communication	Equal variances assumed	0.041	0.839	−0.073	0.94	0.00	0.04
	Equal variances not assumed	–		−0.073	0.94	0.00	0.04

*N* = 575; SD, standard deviation.

#### 3.3.2. Descriptive analysis and multicollinearity test

This study performed a statistical analysis and a validity test for a more comprehensive follow-up regression analysis. Table 4 displays the descriptive statistics, convergent, and discriminant validity results for the survey data. At a certain point, this study ran a multicollinearity test on the data to ensure that it was appropriate for regression analysis. Using SPSS 22.0, the VIF value of all variables was found to be <10, which indicates that the independent variables are not multicollinear, and regression statistical analysis can be conducted. Furthermore, when it comes to the convergent and discriminant validity of all these variables, we used AVE and CR to test whether the variables are of discriminant validity. Based on the results in Table 4, the A.V.E and C.R values are acceptable (AVE > 0.5; CR > 0.7). This result demonstrates that the variables in the theoretical framework are of acceptable discriminant validity.

**Table 4:** Descriptive statistics, convergent, and discriminant validity.

**Variables**	**Mean**	**SD**	**AVE**	**CR**	**1**	**2**	**3**	**4**	**5**	**6**
Customer engagement (1)	4.08	0.52	0.65	0.85	(0.81)					
Transactional psychological contract (2)	3.72	0.67	0.56	0.86	0.498^**^	(0.75)				
Relational psychological contract (3)	4.13	0.52	0.55	0.83	0.657^**^	0.510^**^	(0.74)			
Brand image (4)	4.17	0.53	0.53	0.87	0.676^**^	0.435^**^	0.697^**^	(0.73)		
Perceived quality (5)	4.11	0.59	0.8	0.89	0.472^**^	0.413^**^	0.512^**^	0.521^**^	(0.89)	
Brand communication (6)	4.30	0.50	0.76	0.86	0.526^**^	0.366^**^	0.574^**^	0.568^**^	0.586^**^	(0.87)

*N* = 575; ^**^P < 0.01; SD, standard deviation; AVE and CR provide evidence of the validity of this model following testing; the diagonal value is the square root of AVE.

## 4. Result

**Figure 2:**
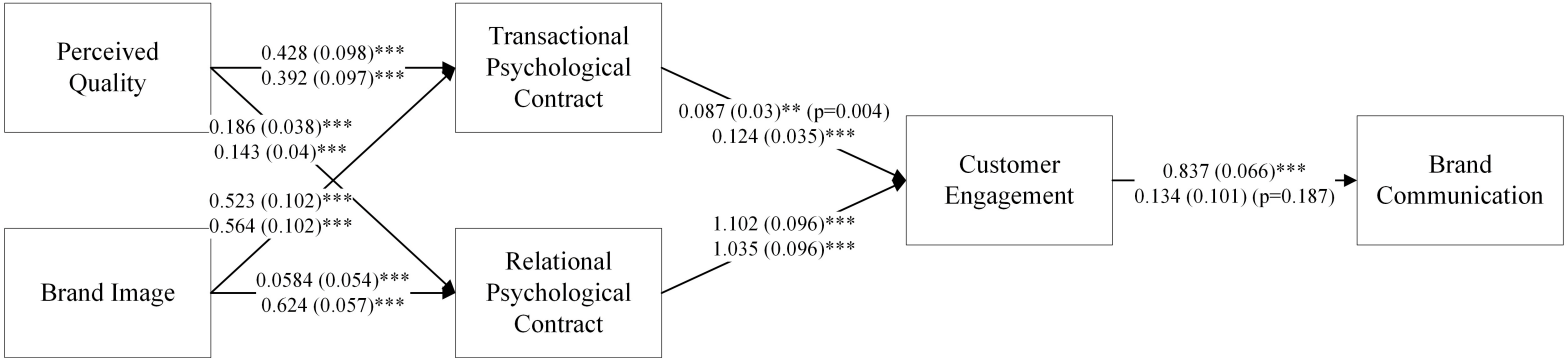
SEM results of the hybrid communication model in utilitarian and hedonic aspects. *N* = 575; ***P < 0.001, **P < 0.01.

In order to test the model proposed in this study, this study depended on SEM through Amos 24.0. All results are displayed in Figure 2.

In this study, gender, age, education, occupation, and salary (monthly) are used as control variables. This research first concentrated on the main effect without control variables, then the control variables were added to the analysis. The results of SEM without control variables have already demonstrated a great degree of model fit. In turn, the results of SEM with control variables showed a better model fit than without controls. The coefficients of each path are shown in Figure 2 and Table 5. Specifically, the above estimates are parameters without controls, and those below are with controls.

**Table 5:** Results of SEM.

**Hypothesized path**	**Model without controls**			**Model with controls**		
	**Path coefficient**	**SE**	**CR**	**Path coefficient**	**SE**	**CR**
**Main effect**						
TPC ← PQ	0.428^***^	0.098	4.353	0.392^***^	0.097	4.025
RPC ← BI	0.584^***^	0.054	10.818	0.624^***^	0.057	10.956
RPC ← PQ	0.186^***^	0.038	4.847	0.143^***^	0.04	3.589
TPC ← BI	0.523^***^	0.102	5.132	0.564^***^	0.102	5.542
CE ← TPC	0.087^**^ (*p* = 0.004)	0.03	2.89	0.124^***^	0.035	3.497
CE ← RPC	1.102^***^	0.096	11.437	1.035^***^	0.096	10.821
BC ← CE	0.837^***^	0.066	12.638	0.134 (*p* = 0.187)	0.101	1.32
**Control effects**						
BC ← Gender	–		1		–	
BC ← Age			1			
BC ← Occupation			1			
BC ← Education			1			
BC ← Salary (monthly)			1			
**Model fit**						
CMIN/DF	3.092			2.423		
RMR	0.028			0.032		
TLI	0.905			0.912		
GFI	0.902			0.907		
CFI	0.915			0.922		
RMSEA	0.06			0.05		

*N* = 575;

*** P < 0.001,

** P < 0.01. PV, perceived value; PI, perceived image; TPC, transactional psychological contract; RPC, relational psychological contract; CE, customer engagement; BC, brand communication; SE, standard error.

It can be observed that these controls have some impact on brand communication. For the last estimate of this model, the main effect of brand communication is now not considered significant (*p* > 0.01). This demonstrates that brand communication, which is of great complexity, is primarily influenced by a variety of factors. Nevertheless, this does not prevent us from analyzing the whole communication mechanism. Table 5 details the results of SEM.

### 4.1. Improving brand communication from the utilitarian and hedonic perspectives

Based on the SEM results, it can be observed that brand communication can be improved through utilitarian and hedonic ways. This fact is supported by the evidence listed later. First, when it comes to the utilitarian aspect, perceived value influences the construction of the transactional psychological contract more than the relational psychological contract (β_*Transaction*_ = 0.392, *P* < 0.001; β_*Relation*_ = 0.143, *P* < 0.001; β_*Transaction*_ > β_*Relation*_). For the customer, the utilitarian aspect of information has always been focused on making decisions and so on. In contrast, from the viewpoint of the hedonic aspect, a perceived image shows more impact on the construction of the relational psychological contract than on the transactional psychological contract (β_*Transaction*_ = 0.564, *P* < 0.001; β_*Relation*_ = 0.624, *P* < 0.001; β_*Transaction*_ < β_*Relation*_). These results led to the identification and support of Hypothesis 1 and its sub-hypotheses.

Moreover, psychological contracts have accelerated the process of achieving customer engagement. Through the results offered by SEM, it can be observed that the psychological contracts, whether transactional or relational, positively influenced customer engagement overall (β_*TM*_ = 0.124, *P* < 0.001; β_*RM*_ = 1.035, *P* < 0.001; β_*TM*_ < β_*RM*_). Thus, Hypothesis 2 and its sub-hypotheses are supported. These findings of estimates present us with a more interesting phenomenon that relational psychological contracts present greater impacts on customer engagement than transactional psychological contracts.

At the last stage of this communication process, customer engagement leads to the improvement of brand communication. Although brand communication was found to be influenced by many factors according to multiple previous studies (Harrison and Windeler, 2020), it is still positively influenced by customer engagement (β_*without controls*_ = 0.837, *P* < 0.001; β_*with controls*_ = 0.134, *P* > 0.01). These analysis results support Hypothesis 3. Consequently, due to the results offered by SEM, it can be concluded that brand communication can be strengthened from both utilitarian and hedonic perspectives.

### 4.2. Mediating effect analysis

In order to test the whole mechanism, the mediation of this theoretical model was tested. According to Preacher and Hayes (2004), the test of the mediation effect has relied on the Bootstrap utilized through SPSS 24.0 PROCESS. The results of this test are displayed in Table 6.

**Table 6:** The chain mediation effect testing based on Bootstrap.

**Independent variable**	**Mediator 1**	**Mediator 2**	**Dependent variable**	**Mediation category**	**CI (95%)**	**Effect**	***T***	**SE**
Perceived quality	Transactional psychological contract	Customer engagement	Brand communication	Direct	(0.2979, 0.4203)	0.3591	11.5207	0.0312
				Indirect	(0.0899, 0.1861)	0.1348	-	0.0243
Brand image	Relational psychological contract			Direct	(0.1504, 0.334)	0.2422	5.18	0.0468
				Indirect	(0.2184, 0.3755)	0.2937	-	0.0401

*N* = 575; SE, represents standard error; CI (95%), represents the confidential interval at 95% level.

Bootstrap is one of the primarily significant methods to identify mediating effects, especially chain mediating effects. In order to test whether there is mediation or not, we shall figure out whether the confidence interval contains 0. The provided results support Hypothesis 4 and its sub-hypotheses. From the perspective of cognition, the transactional psychological contract and customer engagement act as mediators in this mechanism. Through the Bootstrap findings, it can be observed that if one wants to strengthen brand communication in the utilitarian aspect, one should first start from the perceived value and then pass through the transactional psychological contract and customer engagement, ultimately achieving a great degree of brand communication [*CI* (95%)_*direct*_ = (0.2979, 0.4203); *CI* (95%)_*indirect*_ = (0.0899, 0.1861)]. Similarly, from the viewpoint of affection, the results show that the relational psychological contract and customer engagement play a chain mediation role in improving brand communication [*CI* (95%)_*direct*_ = (0.1504, 0.334); *CI* (95%)_*indirect*_ = (0.2184, 0.3755)]. Thus, through this finding, the mechanism by which the hedonic aspect strengthens brand communication was determined.

## 5. General discussion

This study has characterized the mechanism by which brand communication is improved from utilitarian and hedonic perspectives. In this study, the theoretical model was first proposed following a literature review based on the linkage of the practical background. Relying on the questionnaire survey method, this study applied SEM to test the theoretical model. The results of testing demonstrated that all the presented hypotheses were entirely supported. This finding leads to the conclusion that brand communication can be strengthened from utilitarian and hedonic perspectives. However, there are still some remaining aspects that require further discussion.

### 5.1. Theoretical contribution

This study offers some theoretical insights and contributions to the research of brand communication mechanisms. These are outlined as follows. To begin with, this study has established a hybrid model of brand communication mechanism from an information function perspective. The proposition of this hybrid model has filled up the research gap in the marketing and branding research area, as well as contributed to the related studies on customer psychology and behavior. Second, during the process of research, it can be identified that the information function of brands can provide both a utilitarian and a hedonic function to the customers. This is reflected by the chain mediation effect of the psychological contract and customer engagement. Finally, this study has provided some insights into understanding customer behavior in which their psychological aspect has dominated. All theoretical contributions have been organized as follows.

First and foremost, despite this model having been constructed with utilitarian and hedonic aspects, there is an issue in that the function and role of the customer's psychological aspect need to be identified in the digital era. According to prior studies, a variety of scholars have insisted that utilitarian aspects play a critical role, but they have not figured out the specific role of the customer's psychological aspect in the mechanism of brand communication (Hayes et al., 2020; Miao, 2021; Gbadeyan and Deliceirmak, 2022). Based on this theoretical gap, the model provided by this study may offer some explanation. Although it is necessary for many people to depend on the utility of a brand for the utilitarian aspect, the power of the hedonic aspect, that is, the customer's psychological aspect, cannot be neglected. In this model, the findings have offered insights given the fact that if there was a conflict between the utilitarian and hedonic aspects of brand communication, people would yield to their psychological aspects. It can be exemplified that the estimates of their sentiment are all above those of cognition. People need the stimulation of their psychological aspect for enjoyment and sentimental gratification. On the other hand, these dominant effects of the customer's psychological aspect in the process of brand communication also reveal that the function of the brand is mainly to provide a certain hedonic aspect to customers. This relationship is a typical way in which customers connect with brands. The role of sentiment and spirit of customers, therefore, reveals that this consumption is relational. Moreover, this fact reflects that our society may be a relational society, where every individual demands a connection between themselves and others. Consequently, this model provides some solutions for how the firms should make the strategy of brand communication.

Furthermore, despite the fact that the role of the hedonic aspect has taken precedence over that of the utilitarian aspect in brand communication, it can be questioned whether the utilitarian function of information provided by brands has lost its position. For the customers themselves, though a large amount of time spent on media has been filled with “searching for sensitive experiences of brands,” brands not only provide such experiences but also influence the rationale of people (Ong and Yusoff, 2015). Specifically, the utilitarian function of information provided by brands, that is, perceived quality, is still the bedrock demanded by customers. The model developed in this study also confirms the fact that people need information provided by brands to be rational. The improvement of brand communication from the utilitarian perspective cannot be neglected, no matter how dominant the customer's psychological aspect is.

Finally, in terms of brand communication, it can be demonstrated by the SEM results that this variable is of such great complexity that it may be influenced by many factors. Based on the results without the controls, it can be observed that customer engagement has more impact on brand communication (β_*without controls*_ = 0.837, *P* < 0.001). However, when it comes to the status of controls, customer engagement has not played a critical role. For determining whether or not the control variables have a greater influence on brand communication, our study utilized SPSS 24.0 to establish a hierarchy regression model, and the results are depicted in Table 7.

**Table 7:** Hierarchy regression results of control variables and customer engagement on brand communication.

**Variable**	**β**	**SE**
(Constant)	2.173^***^	0.179
Gender	−0.038	0.036
Age	−0.043^**^	0.017
Occupation	0.017	0.016
Education	0.004	0.024
Salary (monthly)	0.06^***^	0.019
Customer engagement	0.495^***^	0.034
*R* ^2^	0.297	
△*R*^2^	0.289	
*F*	39.942	

*N* = 575;

*** P < 0.001;

** P < 0.01.

It can be illustrated that, as mentioned earlier, hypothesis 3 is supported by these regression results. In this regression model, all the control variables were found not to have a significant influence on brand communication, and their β-values are less than those of customer engagement (β_*without controls*_ = 0.495, *P* < 0.001). This result ultimately supports the fact that brand communication is a highly complex variable worthy of further study.

As a result, our findings contribute to the research of brand communication by taking utilitarian and hedonic perspectives. In order to explain the role that the information function played, a hybrid brand communication model has been constructed. The results of this study show that the psychological aspect takes precedence over the rationale of the customer in this modern era.

### 5.2. Practical implication

This research provides not only theoretical contributions to brand communication but also offers practical implications for managing brand information and communication in the post-truth era. Prior studies regarding media and journalism have demonstrated that we are marching into the post-truth era (Waisbord, 2018; Majin, 2021), in which the affection of people takes a dominant position. Based on the definition of the post-truth era from the Oxford Dictionary, the most significant feature of this era is “Relating to or denoting circumstances in which objective facts are less influential in shaping public opinion than appeals to emotion and personal belief.” In addition, it can be observed that brand communication always has a close connection to the media and information from the perspective of its definition. Thus, the characteristics of the post-truth era have inevitably influenced brand communication. This research has revealed that the psychological aspect, that is, the hedonic aspect of customers, has played a significant role in the brand communication mechanism. This results in an important marketing strategy that corporations should pay more attention to customer relationship marketing.

Specifically, when managers make strategies for brand communication, they should consider both utilitarian and hedonic brand offerings. First, the utilitarian function is fundamental, so managers should not neglect this basic function. Thus, in terms of products, firms should focus on improving the quality of products, as well as constructing utilitarian information the brands are delivering. In contrast, in this relational era, according to the characteristics of post-truth, marketing strategy should lay emphasis on the hedonic function that brands convey. When these people start to work on brand communication strategy, they shall put the psychological aspect of brands to the primary position in order to build a sentimental and spiritual image of brands. Therefore, a “warmth” customer relationship can be built and sustained.

## 6. Limitations and future research

Our study has some limitations as the two-fold. On the one hand, as brand communication is of great complexity, this study only explored the impact mechanism of the psychological contract and customer engagement. It is necessary for scholars to discuss and explore the more general factors influencing brand communication. On the other hand, the total number of questionnaires should have been more than 575, which could have been achieved by conducting a larger-scale survey with more questions to resolve certain unknowns.

In terms of future studies, the results of this study provide several insights. Initially, the future study shall pay more attention to the relational demand of customers. The value of relationships has been delivered by this study, and it reveals a future research direction in brand communication. Second, the specific role and function of affection in communication should be more thoroughly discussed in future research. Finally, the results also recall the scholarship to balance the utilitarian and hedonic aspects in the process of brand communication in future.

## Data availability statement

The original contributions presented in the study are included in the article, further inquiries can be directed to the corresponding author.

## Author contributions

LF contributed to the whole process of this article, including proposing research questions and research models, data collecting and processing, and manuscript writing. ML and LT contributed to the revision of this article. All authors contributed to the article and approved the submitted version.
